# Cinema Therapy: From the Silent Film Era to the Present Day

**DOI:** 10.1192/j.eurpsy.2025.207

**Published:** 2025-08-26

**Authors:** M. Poltrum, M. Poltrumn

**Affiliations:** Faculty of Psychotherapy Science, Sigmund-Freud-University, Vienna, Austria

## Abstract

**Abstract:**

Under the rubric of cinema therapy, or movie therapy, research has been undertaken for some time to test the effect of films in different therapeutic settings and with different patient groups. Taking a closer look at the history of film therapy, it becomes apparent that at the time of the silent film, there was already a medical discourse on the effect of films in a therapeutic context. In this lecture, principal philosophical-therapeutic elements of consideration regarding cinema therapy are presented along with historical and current discourses; the most important publications are indicated; and the author’s own cinema-therapeutic models, experiences and reflections are reported. In addition, selected case studies show how films can be utilized in a clinical context and which effects and unwanted side effects result from film therapy. The final focus of the lecture is on films that depict psychotherapy, love films, and films that address the phenomena of intoxication, ecstasy, and addiction, and how these have been applied by the author in the inpatient treatment of addicts.

**Publication:**

Martin Poltrum (2025) Cinema Therapy––The Film as a Medicine. From the Silent Film Era to the Present Day. In: Martin Poltrum et al. (eds.) The Oxford Handbook of Mental Health and Contemporary Western Aesthetics, Oxford: Oxford University Press.

**Disclosure of Interest:**

None Declared

